# Oligometastatic Non-Small Cell Lung Cancer: A Practical Review of Prospective Trials

**DOI:** 10.3390/cancers14215339

**Published:** 2022-10-29

**Authors:** Atallah Baydoun, VeAnn L. Lee, Tithi Biswas

**Affiliations:** 1 Department of Radiation Oncology, University Hospitals Cleveland Medical Center, Cleveland, OH 44106, USA; 2 School of Medicine, Case Western Reserve University, Cleveland, OH 44106, USA; 3 Department of Radiation Oncology, Case Western Reserve University School of Medicine, Cleveland, OH 44106, USA

**Keywords:** NSCLC, oligometastatis, oligoprogression, SBRT

## Abstract

**Simple Summary:**

A significant number of patients diagnosed with non-small cell lung cancer (NSCLC) will have a metastatic Stage IV disease at presentation. Among those, patients with limited number of metastases are referred to as oligometastatic, and their treatment will combine systemic and possible local therapy. The aim of this article is to review the current definition of oligometastatic cancer, a historic perspective of lung cancer leading to modern oligometastatic disease and to present available prospective evidence for treatment of oligometastatic NSCLC. We describe trials exploring role of local therapy in oligometastatic NSCLC with actionable mutation in combination with TKI or without any actionable mutation and in combination with chemo-immunotherapy. We also discuss general treatment approaches adopted based on limited data. Finally, we discuss the on-going clinical trials for oligometastatic and oligoprogressive NSCLC.

**Abstract:**

Oligometastatic non-small cell lung cancer (NSCLC) is an intermediate state between localized and widely metastatic NSCLC, where systemic therapy in combination with aggressive local therapy when feasible can yield a favorable outcome. While different societies have adopted different definitions for oligometastatic NSCLC, the feasibility of curative intent treatment remains a major determinant of the oligometastatic state. The management involves a multidisciplinary approach to identify such patients with oligometastatic stage, including the presence of symptomatic or potentially symptomatic brain metastasis, the presence of targetable mutations, and programmed death-ligand (PD-L1) expression. Treatment requires a personalized approach with the use of novel systemic agents such as tyrosine kinase inhibitors and immune checkpoint inhibitors with or without chemotherapy, and addition of local ablative therapy via surgery or stereotactic radiation therapy when appropriate.

## 1. Introduction

Despite the current advances in lung cancer screening, at least 50% of patients with newly diagnosed non-small cell lung cancer (NSCLC) will present with metastatic disease at diagnosis [1]. Among those patients, approximately 37.5% will present with a limited metastatic state referred to as “oligometastatic” [2], requiring a multidisciplinary treatment approach with systemic agents combined with potential metastasis-directed local ablative therapies [3].

In recent decades, the therapeutic paradigm for oligometastatic NSCLC has evolved significantly. The use of systemic therapy remains the cornerstone of the treatment in stage IV disease. Systemic therapy relied initially on platinum-based doublet chemotherapy [4,5], and nowadays includes tyrosine kinase inhibitors (TKI) for tumors having actionable mutations [6] and the use of immune checkpoint inhibitors (ICI). ICI have been utilized as first line therapy with or without traditional chemotherapy and have achieved around 20% survival at five years [7]. The primary tumor is frequently addressed via surgical resection if appropriate [8], or with definitive radiation therapy. Metastases-directed interventions have switched from surgical resection with post-operative radiation to the employment of only stereotactic radiosurgery (SRS) for brain metastases and stereotactic body radiation therapy (SBRT) for metastatic foci outside the brain [9,10].

To date, there is lack of general consensus on a uniform definition of the oligometastatic NSCLC state, and no single therapeutic perspective has been adopted across institutions. In this article, we present a practical review the current concept of oligometastatic NSCLC, discuss the available published prospective trials, synthetize an initial treatment approach, and highlight currently undergoing several clinical trials and potential future directions.

## 2. Search Strategy

**Figure 1:**
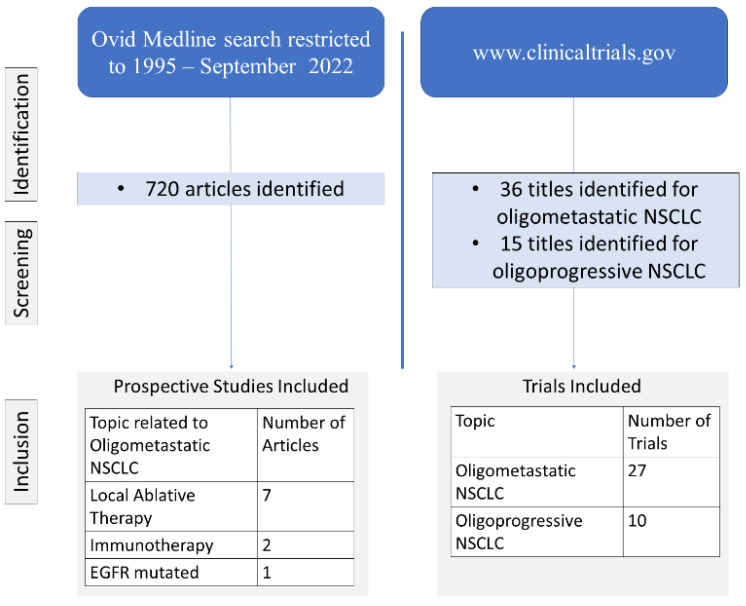
Flowchart for articles and trials inclusion for the current review article. (NSCLC: non-small cell lung cancer, EGFR: epidermal growth factor receptor).

We used a combination of MESH terms and keywords for “oligometastatic NSCLC”, to perform a search in Ovid-MEDLINE limited to the publication years from 1995 till 1st, September 2022. We first classified articles by date of publication and reviewed the full texts of the relevant titles. We then sorted out articles by type of study in order to delineate the current treatment approaches and the data from recent prospective trials for detail description in the current review article. Finally, we performed a search at ClinicalTrials.Gov (access date 15 August 2022) in order to map out the currently undergoing trials for oligometastatic NSCLC to provide detailed description of on-going clinical trials on this topic. The flowchart for articles and trials inclusion is featured in Figure 1.

## 3. The Current Concept of Oligometastatic NSCLC

**Figure 2:**
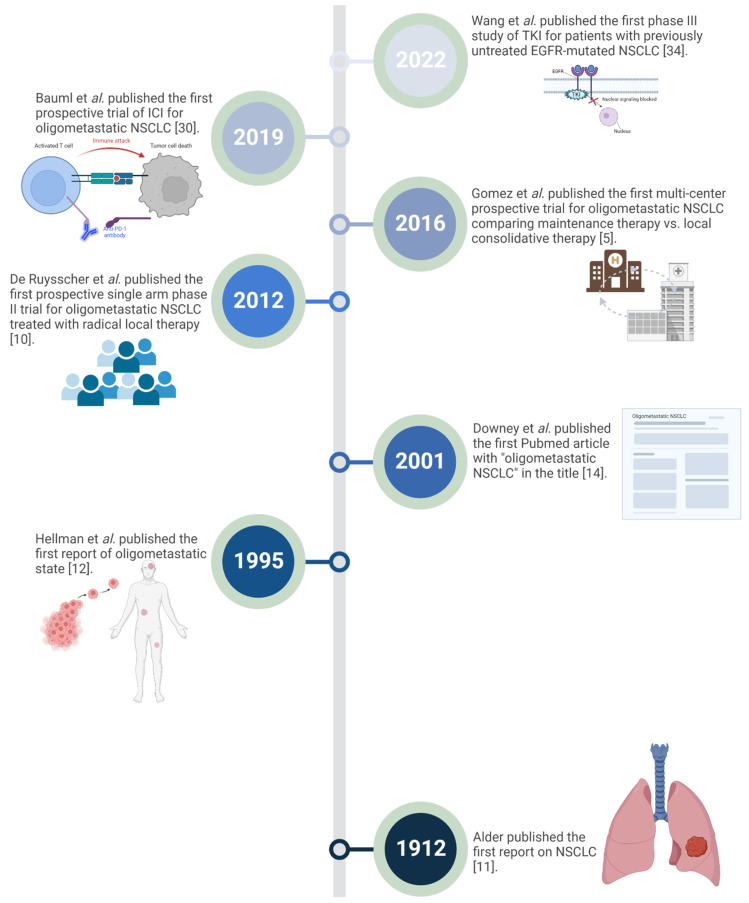
Historical Landmarks in Oligometastatic NSCLC. (NSCLC: Non-small cell lung cancer; ICI: Immune checkpoint inhibitors; TKI: Tyrosine kinase inhibitors; EGFR: Epidermal growth factor receptor).

In a book entitled “Primary Malignant Growths of the Lungs and Bronch: A Pathological and Clinical Study “published in 1912, Adler summarized the available data about primary lung cancer at that time, consisting of 374 cases, the oldest of which goes back to 1852 [11]. Eight decades later, in 1995, Hellman et al. introduced the concept of oligometastasis as an intermediate status between localized disease and widespread distant metastasis [12]. Six years later, the term “oligometastases” appeared for the first time in the PubMed titles in 2001 [13], when Downey et al. reviewed the role of resecting a “finite number” of extrathoracic sites in patients with metastatic NSCLC [14]. Following Downey’s report, the number of research manuscripts addressing oligometastasis started to increase steadily [13] and grew exponentially in the last decade [15] with the advancement of novel systemic agents, and advanced radiation technology such as SRS, and SBRT treatment approaches. A historical perspective of oligometastatic NSCLC entity is illustrated in Figure 2. For practical purpose, we will use SRS in the rest of this article to refer to the focal radiation therapy delivered to the brain for a non-palliative approach in five or fewer sessions. When extra-cranial sites are targeted with the same scheme, radiation therapy will be referred to by SBRT.

The three main challenges for consolidating a uniform definition of the oligometastasis concept lay in delineating (1) the extent of metastatic burden for which a patient is considered to be in oligometastatic state, (2) the impact of the timing between the diagnosis of the primary lesion and the diagnosis of the metastasis, and (3) the response of the metastatic disease and the primary tumor to the systemic therapy. In terms of metastatic burden, different views have surged over the years about the number of metastatic lesions that would define an oligometastatic state. The most commonly used definitions were ≤3 or ≤5 distant metastatic regions [16,17]. On the other hand, an ESTRO-ASTRO consensus published in 2020 [18] outlined four categories of oligometastatic cancer dependent on the timing of appearance of metastatic lesions appearance and the response to systemic therapy. These categories included (1) synchronous oligometastatic disease where oligometastasis and the primary tumor are detected concurrently at the time of diagnosis, (2) metachronous oligometastatic or oligo-recurrent disease where oligometastatic lesions are detected three months after the initial diagnosis while the primary tumor is controlled, (3) oligo-progressive metastatic disease where progression after systemic therapy occurs in a limited number of metastases, and (4) oligo-persistent metastatic disease where oligo-metastases persist after systemic therapy [18]. Nevertheless, a universal definition of oligometastatic disease has not been adopted yet, and discrepancy regarding the number of organs involved, the volume of the metastasis, and the response of the primary tumor persists, in addition to the patient’s performance status and the likelihood of tolerating local ablative therapies [3]. 

The definition of the oligometastatic state for patients with NSCLC also presents a significant degree of heterogeneity across different institutions. In a systematic review published in 2019 by Schanne et al. [8], the number of metastases that defined an oligometastatic NSCLC varied between 1–8 across studies, nearly 60% of the studies did not include a formal definition of the oligometastatic state, and around 90% of the patients were treated with local ablative therapy to only a single metastasis [8]. Particularly for NSCLC, a further degree of inconsistency in the definition of the oligometastatic state lies in whether or not the involved mediastinal lymph nodes are considered a site of metastases [19]. In a multidisciplinary expert opinion initiated by the European Organization Research and Treatment of Cancer: Lung Cancer Group (EORTC-LCG), the proposed definitions for synchronous oligometastatic NSCLC included 1 metastasis in 1 organ, 1–3 metastases, ≤3 metastases including mediastinal lymph nodes after systemic therapy, and 3–5 metastases [19]. These variations led to the elaboration of a consensus from different societies including EORTC-LG and the International Association for the Study of Lung cancer published in 2019 [20] that proposed a maximum of 5 metastases and 3 organs as a definition synchronous oligometastatic NSCLC [20]. Pulmonary metastases were counted as a metastatic site while mediastinal lymph nodes were not [20]. The consensus highlighted the fact that the definition of synchronous oligometastatic disease is relevant only when a radical treatment is feasible and thus patients with meningeal, pericardial, pleural, mesenteric, or bone marrow involvement were excluded from the definition [20]. A summary of these definitions is provided in Table 1.

Based on the available information, American Joint Commission on Cancer (AJCC) in the 8th edition incorporated a distinct sub-category under stage IV disease to indicate better prognostication in this subset of patients with oligometastatic disease [21]. According to the definition provided by AJCC, presence of single metastasis in a single organ at the time of initial diagnosis will be designated as “M1a” or stage IVA [21]. It remains be seen whether this staging category will change in future as more information emerges through future clinical trials. 

As for the oligo-progressive state, it usually refers to the subset of patients with metastatic NSCLC who, after achieving an objective response to systemic therapy, exhibit disease progression in a single or few limited metastatic sites [22]. The American Radium Society defined oligo-progressive disease as the increase in size and/or avidity of up to 3 metastatic area compared to the initiation of systemic therapy, in patients who are receiving or have finished systemic therapy [23]. The ESTRO-ASTRO characterized oligo-progression by up to 5 metastatic lesions that progressed in the settings of a widespread but stable metastatic disease [18]. Finally, a Canadian consensus suggested that progression in up to 4 discrete lesions amenable to local ablative therapy after a period of stable disease, partial response, or complete response can be referred to as oligo-prgression [24]. Differences in tumor microenvironment are thought to cause the selective progression in only some of the metastatic sites in oligo-progressive disease, but the exact etiology is not well understood yet.

## 4. Prospective Trials for Local Ablative Therapy in Oligometastatic NSCLC

The first prospective trial for oligometastatic NSCLC was a prospective single-arm phase II trial published in 2012 by De Ruysscher et al. [10] who reported 1-year progression free survival (PFS) of 51.3% in 39 patients with synchronous oligometastatic NSCLC [10]. While the inclusion criteria allowed for five metastases at the time of diagnosis, 34 (87.2%) patients had one metastasis, 4 (10.3%) had two metastases, and 1 (2.6%) had three metastases [10]. There were no patients with more than three oligometastases, the most frequent site of treated metastasis was the brain with 17 (43.9%) patients, and local ablative therapy included a combination of surgical resection and SRS for brain metastases or conventional post-operative radiotherapy for incompletely resected extracranial metastases [10]. In 2014, Collen et al. [25] published a prospective phase II study that recruited 26 patients with oligometastatic NSCLC defined as five or less, intracranial or extracranial, metastatic lesions to receive SRS/SBRT to primary tumor and metastatic locations [25]. Seven patients had metachronous oligometastatic NSCLC, while 19 had synchronous presentation [25]. SRS and SBRT were delivered uniformly as 50 Gray (Gy) in 5 fractions, and the 1-year PFS and 1-year overall survival (OS) were 45% and 67%, respectively [25]. Petty et al. published in 2018 the long-term outcome of a phase II, single-arm trial during which patients with oligometastatic NSCLC with a maximum of five metastases and no more than three active extracranial lesions were treated with consolidative radiation therapy after achieving a partial or stable response with platinum-based chemotherapy [26]. Radiotherapy consisted of SBRT or a conventional fractionation regimen of 60 Gy in 30 fractions [26]. This study was closed early due to slow accrual, and it achieved a median PFS of 11.2 months and a median OS of 28.4 months [26]. Iyengar et al. published in the same year the results of a single institution phase II study, during which patients with limited metastatic disease following the completion of first line chemotherapy were randomized to receive maintenance chemotherapy alone or SBRT followed by maintenance chemotherapy [4]. PFS was improved (*p* = 0.01) in the SBRT followed by the maintenance chemotherapy arm (9.7 months) compared to the maintenance chemotherapy alone arm (3.5 months) [4]. Interestingly, six patients in the SBRT arm and five patients in the maintenance chemotherapy alone arms had brain metastases treated prior to enrollment, but the number of brain lesions was not associated with PFS or OS [4]. In another phase II single-arm study, Arrieta et al. enrolled 37 patients with synchronous oligometastatic NSCLC, defined as the presence of five or less metastases [27]. Patients who had stable or partial response after four initial cycles of systematic therapy were treated with local ablative therapy to the primary site and metastases and a median PFS of 23.5 months was achieved [27]. The study included patients with epidermal growth factor receptor (EGFR) and anaplastic lymphoma kinase (ALK) mutations, and 11 patients received TKI [27].

The first multicenter prospective randomized trial for patients with oligometastatic NSCLC was published in 2016 by Gomez et al. who randomized patients without disease progression after first-line systemic therapy to maintenance therapy versus local consolidative therapy consistent of surgery, radiotherapy, or both [5]. At a median follow-up of 12.39 months, the median PFS was 11.9 months in the local consolidative therapy group versus 3.9 months in the maintenance therapy group (Hazard Ratio (HR) 0.35, log-rank *p* = 0.0054), and the study was closed earlier due to the significant improvement with local therapy [5]. The long-term results of this trial were reported in 2019 [28]. With a median follow-up time of 38.8 months, there was a persistent benefit in PFS (*p* = 0.022) with a median PFS of 14.2 months in the local consolidative therapy arm versus 4.4 months in the maintenance therapy group arm [28]. A statistically significant difference was also seen in OS (*p* = 0.017), with a median OS of 41.2 months in the local consolidative therapy arm versus 17.0 months in the maintenance therapy group arm [28]. In the SABR-COMET trial [29], 18/99 enrolled patients had oligometastatic NSCLC and a significant difference in 5-year OS (stratified log-rank, *p* = 0.006) was noted between SRS/SBRT and standard of care arm (42.3%) versus standard of care alone arm (17.7%) [29].

## 5. Immunotherapy in Oligometastatic NSCLC

Beyond local ablative therapy, the development of ICI constituted a landmark improvement in the treatment model for metastatic NSCLC including oligometastatic disease. In 2019, Bauml et al. published the results of a single-arm phase II trial that enrolled patients with metachronous or synchronous oligometastatic NSCLC to receive pembrolizumab after completion of local ablative therapy [30]. Any form of ablative local therapy was permitted and at least 53% of the patients had received chemotherapy prior to enrollment [30]. Oligometastatic state was defined as the presence of four or less metastatic lesions, and median PFS was 19.1 and 18.7 months after the start of the ablative local therapy and the start of pembrolizumab therapy, respectively [30]. In another prospective trial, 76 patients with recurrent metastatic NSCLC were randomized to pembrolizumab alone (control arm) versus SBRT followed by pembrolizumab (experimental arm) [31]. Median PFS was 7.6 months in the experimental arm versus 1.9 months in the control arm (HR 0.71, *p* = 0.19), and OS was 15.9 months in the experimental arm versus 7.6 months in the control arm (HR = 0.66, *p* = 0.16) [31]. Most of the other published studies included retrospective data analysis showing superiority when local consolidative therapy is used in conjunction with ICI. For example, a retrospective review performed by Chen et al. included 231 patients with synchronous oligometastatic NSCLC treated with first-line pembrolizumab with or without chemotherapy, among which 76 patients received local consolidative therapy after first line systemic therapy. PFS and OS in patients receiving local consolidative therapy (14 and 30.7 months, respectively) were significantly better (*p* = 0.016 and *p* = 0.011, respectively) compared to patients receiving only first-line pembrolizumab with and without chemotherapy (10.1 and 22 months, respectively) [32].

## 6. Oligometastatic EGFR Mutated NSCLC

Since the availability of several TKI directing at EGFR mutant NSCLC, these drugs have become the primary modality of treatment in stage IV NSCLC with EGFR mutation. Generally, there is interest in combining local consolidative therapy with TKI in these patient populations. Initial retrospective studies have suggested an improved outcome with the combination of TKI and local consolidative therapy. For example, a retrospective review published in 2018 included 145 patients with oligometastatic EGFR-mutant NSCLC divided into three groups: (1) 51 patients with consolidative local ablative therapy to all oligometastatic sites, (2) 55 patients with consolidative local ablative therapy to either primary tumor or oligometastatic sites, and (3) 39 patients who did not receive local therapy [33]. All patients were treated with EGFR-TKI. There was statistically significant improvement seen in OS for patients treated with consolidative local therapy to all oligometastatic sites (Group 1) compared to the group of patients who did not receive local therapy (Group 3) and to the group who received local therapy to either primary tumor or oligometastatic site (Group 2) [33]. More recently, the SBRT in newly diagnosed advanced staged lung adenocarcinoma (SINDAS) trial was the first published phase III trial evaluating the role of TKI with or without radiotherapy in patients with EGFR driven oligometastatic NSCLC [34]. In SINDAS, patients with newly diagnosed metachronous oligometastatic NSCLC without brain metastases, with up to five extra-cranial metastases, and no more than two metastatic lesions per organ were randomized to receive first-generation TKI (gefitinib, erlotinib, or icotinib) alone or first-generation TKI with upfront radiotherapy defined as 25–40 Gy in 5 fractions depending on the tumor size and location [34]. Interim analysis at 68% accrual included a total of 136 patients. Patients treated with upfront TKI and radiation therapy had a favorable PFS (20.2 months versus 12.5 months, *p* < 0.001) and OS (25.5 months versus 17.4 months, *p* < 0.001) compared to patients treated with TKI only [34].

## 7. Ongoing Trials

Table 2 summarizes the currently ongoing clinical trials for oligometastatic NSCLC. Among the 27 listed trials, one trial is evaluating SRS/SBRT to 3–10 oligometastatic lesions [35], one trial is evaluating SBRT for 1–6 extracranial sites followed by Durvalumab and Tremelimumab [36], two trials did not provide a formal definition of the oligometastatic state [37,38], and the rest of the 22 trials have defined the oligometastatic state heterogeneously by including up to three or five lesions. A total of 11 (41%) studies are two-arm prospective trials [39,40,41,42,43,44,45,46,47,48,49]. Local therapy via SBRT/SRS is being evaluated in 13 (48%) studies [35,36,39,47,50,51,52,53,54,55,56,57]. Many of the two-arm trials mainly evaluate the addition of ablative local therapy to the standard medical therapy. For example, OMEGA trial is a randomized phase III trial comparing standard medical treatment versus local ablative therapy in patients with synchronous or metachronous oligometastatic NSCLC [39]. TARGET-02 is also comparing standard medical treatment versus local consolidative radiation therapy and standard medical treatment in patients with previously treated oligometastatic NSCLC [43]. Similarly, SARON is comparing systemic therapy to systemic therapy followed by SBRT/SRS [45]. Another phase II trial is comparing SBRT to conventional radiotherapy for oligometastatic NSCLC [44]. TARGET-01 is comparing continuation of TKI therapy versus continuation of TKI therapy in combination with local consolidative radiation [40]. Another phase II study is comparing Lazertinib versus Lazertinib plus SBRT/SRS in patients with EGFR-mutated synchronous oligometastatic NSCLC [47]. On the other hand, the OITROLC study is evaluating whether the sequence of treatment affects the response rate by comparing upfront concurrent chemoradiotherapy followed by chemotherapy versus initial radiotherapy followed by concurrent chemoradiotherapy [41]. The primary evaluated outcome is PFS in 15 (56%) trials [35,40,42,44,46,47,48,49,50,52,54,58,59,60], and OS in 6 (22%) studies [39,43,45,55,60,61]. Finally, the first large co-operative group trial, LU002, is a phase II/III randomized trial comparing standard chemotherapy or immunotherapy followed by local consolidative or ablative therapy versus none in oligometastatic NSCLC is currently ongoing [60]. If positive, this trial will guide on incorporating the use of ablative local therapy across both academic and community treatment centers. 

Table 3 lists the 10 currently ongoing trials for oligoprogressive NSCLC. Among these trials, three trials are evaluating treatment for EGFR mutated oligoprogressive NSCLC with TKI combined with local therapy [53,62,63]. ICI combined with SBRT/SRS is being evaluated in two trials [64,65]. The definition of the oligometastatic state is also heterogeneous, though no more than five oligometastatic extracranial lesions are included. OS is the primary outcome in only one trial [66], while most of the remaining trials evaluated PFS. One phase II trial is evaluating whether the sequence of ablative therapy and TKI therapy (ablative therapy followed by Osimertinib versus Osimertinib followed by ablative therapy) affects the PFs [53]. Two phase II trials are evaluating the addition of SBRT/SRS to standard medical therapy [66,67], and one phase II trial (HALT) is comparing continued TKI therapy versus continued TKI therapy in combination with SBRT [68]. 

## 8. Oligometastatic NSCLC: Initial Therapeutic Approach

Oligometastatic NSCLC is often perceived as a favorable therapeutic opportunity during which aggressive therapy can yield favorable outcomes approaching those of patients with non-metastatic NSCLC. Under this perspective, the management of patients with oligometastatic NSCLC requires a multi-disciplinary approach. For patients with initial presentation suggestive of metastatic NSCLC, the primary objective of the diagnostic and therapeutic assessment is to determine whether the presentation can be appraised as oligometastatic. Such determination can be ideally made based on the EORTC-LG and the International Association for the Study of Lung cancer consensus [20], the patient’s performance status, comorbid conditions, and the feasibility of a definitive intent treatment. In addition, the presence or not of actionable mutations and the programmed death-ligand (PD-L1) expression status should be determined. 

In patients deemed to be in an oligometastatic state, participation in clinical trials should always be considered. Otherwise, the sequencing of the treatment modalities can be initiated with upfront systemic therapy except for intracranial metastasis needing early intervention based on available literature as described above. In patients with EGFR driven oligometastatic NSCLC without brain metastases, treatment can be a combination of upfront TKI combined with local ablative therapy as utilized in the SINDAS trial [34]. As for patients with brain metastases at initial presentation, the optimal approach remains indeterminate [69], but can possibly include an initiation of third generation TKI based on two trials showing good central nervous system response rate [70,71]

**Figure 3:**
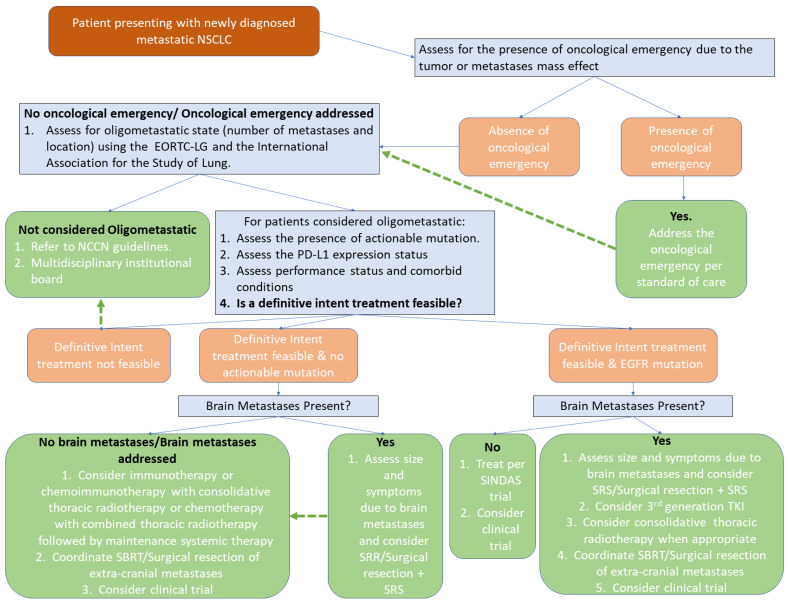
Flowchart for the suggested initial approach in patients presenting with metastatic (NSCLC: non-small cell lung cancer; EORTC-LG: the European Organization Research and Treatment of Cancer: Lung Cancer Group; NCCN: National Comprehensive Cancer Network; PD-L1: programmed death-ligand; EGFR: epidermal growth factor receptor).

For oligometastatic NSCLC patients without actionable mutation, treatment would include first-line systemic therapy, followed by local consolidative therapy via surgical resection or SRS/SBRT in case of no progression. Depending on patient comorbidities and PD-L1 status, immunotherapy or chemoimmunotherapy can also be considered a first-line treatment for systemic therapy option [69]. Such an approach would be similar to the treatment paradigm in the trials of Gomez et al. [28] and Iyengar et al. [4]. Finally, the total treatment duration with ICI and targeted therapy remains also unknown. Different strategies include treatment until response, progression, or significant toxicity have been documented [72], and these are usually employed through institutional practice and adapted on a patient-by-patient basis. A workflow for the initial approach is presented in Figure 3.

## 9. Conclusions

Oligometastatic NSCLC remains a poorly defined entity, and this is reflected in the heterogeneity of the oligometastatic state definition among different societies. This heterogeneity also led to the adoption of different inclusion criteria for the prospective trials, and it renders the projection of prognostic information from the trials to a real-world patient rather challenging.

In the current era of advanced diagnostic modalities, further international multi-disciplinary effort is still needed in order to have a consensus definition of the oligometastatic state in NSCLC. Limited prospective phase II studies have shown encouraging results of adding local ablative therapy using SBRT and SRS to initial chemotherapy, ICI, and TKI. The currently ongoing trials are expected to provide further evidence regarding the optimal treatment strategies. Future multi-omics studies will be required to personalize therapy via accurate prognostication and prediction studies.

## Figures and Tables

**Table 1:** Oligometastatic State Definition for NSCLC.

Society—Year of Publication	Number of Metastatic Sites (Agreement)	Number of Organs Involved (Agreement)	Mediastinal Lymph Node Inclusion (Agreement)	Pulmonary Metastases Status (Agreement)
EORTC-LCG * 2019	1 (12%)	1 (24%)	Yes (72%)	Yes (87%)
	≤3 (42%)	2 (31%)	No (22%)	No (10%)
	>5 (17%)	3 (25%)	N/A (6%)	N/A (3%)
EORTC-LG and the International Association for the Study of Lung cancer 2019	≤5	≤3	Not included in Metastatic Sites Count	Included in Metastatic Sites Count

* EORTC-LCG: European Organization Research and Treatment of Cancer: Lung Cancer Group. NSCLC: Non-small cell lung cancer. For simplification, the answers with the highest three agreements were included for the EORTC-LCG definition.

**Table 2:** Ongoing Clinical Trials for Oligometastatic NSCLC.

Study Title and Number	Treated Condition	Oligometastatic Definition	Study Design	Treatment Scheme	SBRT/SRS	Surgical Excision	Number of Participants	Primary Outcome	Start Date
Toripalimab in Combination With Platinum-based Chemotherapy for Mutation-negative Stage IV Oligometastatic NSCLC NCT05055583	Mutation-negative histologically or cytologically confirmed oligometastatic NSCLC	1–3 and only 1 other organ excluding the primary organ involved	Single-arm prospective phase II study	Toripalimbab in combination with neoadjuvant platinum-based chemotherapy followed by surgery	N	Y	30	PFS	August 2021
OMEGA, Local Ablative Therapy in Oligometastatic NSCLC (OMEGA) NCT03827577	Synchronous or metachronous pathologically confirmed oligometastatic NSCLC	1–3	Two-arm prospective phase III study	Standard medical treatment *	SBRT/SRS * (N/A)	Y	195	OS	October 2019
				Surgery and/or local therapy radiation					
Prospective Trial of Induction Immunotherapy in Locally Advanced or Oligometastatic NSCLC Without a Primary Curative Option (KOMPASSneo) NCT04926584	Histologically or cytologically confirmed locally advanced or oligometastatic NSCLC that cannot undergo initial curative treatment due to large tumor size or functional reasons	Not provided	Prospective observational study	Induction immuno-chemotherapy or immunotherapy * prior to resection or definitive chemoradiotherapy *	N	N	50	Completion of definitive therapy	December 2017
A Trial of SHR-1210 (an Anti-PD-1 Inhibitor) in Combination With Hypofraction Radiotherapy in Patients With NSCLC NCT03557411	Previously treated oligometastatic NSCLC	1–5	Single-arm prospective phase II study	SHR-1210 in combination with hypofractionated radiotherapy	N	N	42	Clinically significant toxicity	July 2018
								PFS	
Immunotherapy, Chemotherapy, Radiotherapy and Surgery for Synchronous Oligo-metastatic NSCLC (CHESS) NCT03965468	Histologically confirmed synchronous oligometastatic NSCLC	1–3 At least 1 must be extra-cerebral	Single-arm prospective phase II study	Induction with Durvalumab, platinum-based including Carboplatin and Paclitaxel, and SBRT followed by definitive local therapy * and continuation of Durvalumab until disease relapses or up to 1 year	SBRT/SRS (max. 10 F over 2 weeks)	Y *	47	PFS	November 2019
Local Consolidative Radiation Therapy Plus TKI Versus TKI Alone in Driver Mutated OM-NSCLC (TARGET-01) NCT05277844	Pathologically confirmed driver mutated oligometastatic NSCLC	1–5 and excluding the primary tumor and regional nodes 1–3 in 1 organ	Two-arm prospective phase II study	Continuation of TKI therapy only	SBRT/SRS * (N/A)	N	106	PFS	November 2019
				Continuation of TKI therapy in combination with local consolidative radiation therapy					
The Optimal Intervention Time of Radiotherapy for Oligometastatic Stage IV Non-small Cell Lung Cancer (NSCLC) (OITROLC) NCT02076477	Pathologically or cytologically confirmed oligometastatic NSCLC	1–5	Two-arm prospective phase III study	Initial concurrent chemoradiotherapy followed by chemotherapy (Docetaxel, Pemetrexed, or Cisplatin)	N	N	420	Short-term effects RR	January 2014
				Neoadjuvant chemoradiotherapy followed by concurrent chemoradiotherapy					
The Value of Radiotherapy in the Oligometastatic Non-squamous Non-small Cell Lung Cancer With Clinical Benefits From Erlotinib as Second-line Treatment (ROLE) NCT01796288	Histologically or cytologically confirmed non-squamous oligometastatic NSCLC and received second-line erlotinib treatment	1–5 1–4 in 1 organ	Two-arm prospective phase II study	Erlotinib only	N	N	200	PFS	October 2012
				Erlotinib simultaneously with radiotherapy					
A Trial Evaluating Stereotactic Radiotherapy Plus Durvalumab Continuation for Patients With NSCLC Metachronous Oligometastatic Disease Under Durvalumab Consolidation Following Chemoradiation (SILK BM) NCT03955198	Metachronous oligometastatic NSCLC patients under durvalumab consolidation following chemoradiotherapy for previous stage III NSCLC	1–5 in 1–3 organs and less than 4 cm	Single-arm prospective phase II study	SBRT/SRS in combination with Durvalumab	SBRT/SRS (N/A)	N	50	PFS	May 2021
Standard Maintenance Therapy (SMT) vs. Local Consolidative Radiation Therapy and SMT in OM-NSCLC (TARGET-02) NCT05278052	Pathologically confirmed and previously treated oligometastatic NSCLC	1–5 and excluding the primary tumor and regional nodes 1–3 in 1 organ	Two-arm prospective phase III study	Standard medical treatment	N	N	190	OS	April 2020
				Local consolidative radiation therapy and standard medical treatment					
A Study of TQB2450 Injection Combined With Stereotactic Body Radiation Therapy (SBRT) in Subjects With Advanced Oligometastatic Non-Small Cell Lung Cancer NCT04306926	Histologically or pathologically confirmed advanced oligometastatic NSCLC intolerable to first-line chemotherapy	1–5	Single-arm prospective phase II study	SBRT followed by TQB2450	SBRT*	N	59	PFS	March 2020
Phase II Trial of SBRT Compared With Conventional Radiotherapy for Oligometastatic Non-Small Cell Lung Cancer NCT02975609	Histologically confirmed, received chemotherapy with response, oligometastatic NSCLC	1–5 less than 5 cm	Two-arm prospective phase II study	Platinum-based chemotherapy followed by conventional fractionated radiotherapy	SBRT (30 Gy/3–5 F)	N	100	PFS	November 2016
				Platinum-based chemotherapy followed by SBRT					
Osimertinib and Locally Ablative Radiotherapy in Patients With Synchronous Oligo-metastatic EGFR Mutant NSCLC (STEREO) (STEREO) NCT04908956	Histologically confirmed EGFR-mutant synchronous oligometastatic NSCLC	1–5 excluding primary tumor	Single-arm prospective phase II study	Osimertinib in combination with SBRT/SRS	SBRT/SRS (max. 5 F)	N	60	Safety of Osimertinib and SBRT	December 2021
Concurrent and Non-concurrent Chemo-radiotherapy or Radiotherapy Alone for Patients With Oligo-metastatic Stage IV Non-small Cell Lung Cancer (NSCLC) NCT01282450	Histologically or cytologically confirmed oligometastatic NSCLC	1–4	Single-arm prospective phase II study	Radical radiotherapy	N	Y *	60	OS	May 2006
Phase Ib Study of Stereotactic Body Radiotherapy (SBRT) in Oligometastatic Non-small Lung Cancer (NSCLC) With Dual Immune Checkpoint Inhibition NCT03275597	Histologically or cytologically confirmed and previously received chemotherapy oligometastatic NSCLC	1–6 extracranial sites Gross tumor volume of less than 8 cm	Single-arm prospective phase I study	SBRT followed by Durvalumab and Tremelimumab	SBRT (30–50 Gy/5 F)	N	17	Safety and tolerability of treatment	February 2018
Stereotactic Ablative Radiotherapy for Oligometastatic Non-small Cell Lung Cancer (SARON) NCT02417662	Histologically or cytologically confirmed oligometastatic NSCLC	1–5 in 1–3 organs	Two-arm prospective phase III study	Systemic anti-cancer therapy	SBRT/SRS (N/A)	N	340	OS	August 2016
				Systemic anti-cancer therapy followed by SBRT/SRS					
Local Non-salvage Radiotherapy for Synchronous Oligometastatic Non-small-cell Lung Cancer. NCT03119519	Histologically or cytologically confirmed synchronous oligometastatic NSCLC	1–5 excluding primary tumor	Two-arm prospective phase II study	Platinum-based chemotherapy or Geffitinib or Erlotinib followed by 3D CRT or IMRT	N	N	148	PFS	December 2017
				Platinum based chemotherapy or Geffitinib or Erlotinib only					
Consolidation Conventional Radiotherapy + Stereotactic Body Radiotherapy at 3 Months After First-line Chemotherapy in Stage IV Oligometastatic Non-small Cell Lung Cancer NCT04758481	Oligometastatic NSCLC	3–10	Single-arm prospective phase I/II study	Platinum-based chemotherapy followed by local radiotherapy then SBRT/SRS then maintenance local therapy	SBRT/SRS (30 Gy/1 F, or 50–60 Gy/3–5 F)	N	20	Acute and late toxicity PFS	May 2021
The Safety and Efficacy of First-line Lazertinib and Locally Ablative Radiotherapy in Patients With Synchronous Oligo-metastatic EGFR-mutant Non-small Cell Lung Cancer NCT05167851	Histologically confirmed synchronous EGFR-mutant oligometastatic NSCLC	1–5 excluding primary tumor	Two-arm prospective phase II study	Lazertinib in combination with SBRT/SRS	SBRT/SRS (max. 5 F)	N	68	PFS	December 2021
				Lazertinib only					
Sintilimab After Stereotactic Ablation Brachytherapy for Refractory Oligometastatic Non-Small Cell Lung Cancer NCT04486287	Refractory oligometastatic NSCLC with failed second-line systemic therapy	Not provided	Single-arm prospective phase II study	Stereotactic ablation brachytherapy followed by Sintilimab	N	N	44	ORR	September 2020
Chemotherapy Combination With Local Radiotherapy and rhGM-CSF for Oligometastatic Stage IV NSCLC Patients (CRAGMOLC) NCT03489616	PR or SD after first-line chemotherapy oligometastatic NSCLC	2–5 1 distant lesion outside the radiation site	Two-arm prospective study	Single agent chemotherapy in combination with local radiotherapy followed by rhGM-CSF	N	N	45	PFS	January 2018
				Single agent chemotherapy only					
Durvalumab Combined With Chemotherapy and Stereotactic Body Radiotherapy (SBRT) in Patients With Oligometastatic Non-small Cell Lung Cancer (NSCLC) NCT04255836	Histologically confirmed oligometastatic NSCLC	1–5 in 1–3 organs	Single-arm prospective phase II study	Durvalumab combined with Paclitaxel + Carboplatin OR Pemetrexed + cisplatin followed by SBRT/SRS	SBRT/SRS (50–60 Gy/1–10 F)	N	35	PFS	September 2020
Radical Treatment of Synchronous Oligometastatic Non-Small Cell Lung Carcinoma NCT02805530	Pathologically confirmed synchronous oligometastatic advanced NSCLC	1–5	Single-arm prospective study	EGFR/TKI therapy * or platinum-based chemotherapy followed by radical local therapy	SBRT/SRS (N/A)	Y *	25	OS	June 2015
Chest Lymph Node Sampling in Patients With Advanced Lung Cancer to be Treated With Curative-intent Radiation Treatment NCT04852588	Oligometastatic NSCLC	1–5 excluding primary tumor	Single-arm prospective study	Endobronchial ultrasound-guided transbronchial fine needle aspiration (EBUS-TFNA) or transesophageal ultrasound-guided fine needle aspiration (EUS-FNA) followed by SBRT/SRS	SBRT/SRS (N/A)	N	29	Changes to treatment plan	November 2021
Surgery and/or Radiation Therapy or Standard Therapy and/or Clinical Observation in Treating Patients With Previously Treated Stage IV Non-small Cell Lung Cancer NCT01725165	Pathologically confirmed previously treated oligometastatic NSCLC	1–3	Two-arm prospective phase II study	Surgical removal and/or local radiation therapy	N	Y *	94	PFS	November 2012
				Standard medical treatment or clinical observation per physician choice with choice of crossing to experimental arm					
Maintenance Systemic Therapy Versus Local Consolidative Therapy (LCT) Plus Maintenance Systemic Therapy for Limited Metastatic Non-Small Cell Lung Cancer (NSCLC): A Randomized Phase II/III Trial (NRG-LU002) NCT03137771	Patients with histologically confirmed metastatic NSCLC with after first-line/induction systemic therapy and no evidence of progression and limited (≤3 discrete sites) metastatic disease, all of which must be amenable to SBRT +/− Surgery	1–3	Phase II/III randomized prospective study	Maintenance systemic therapy alone	SBRT	Y	378	PFS for the phase II	April 2017
				SBRT or SBRT and Surgery to all sites of metastases (≤3 discrete sites) plus irradiation (SBRT or hypofractionated RT) of the primary site followed by maintenance systemic therapy.				OS for the phase III	
Stereotactic Body Radiation Therapy After Surgery in Treating Patients With Stage III–IV Non-small Cell Lung Cancer NCT01781741	Histologically confirmed oligometastatic NSCLC	1–3	Single-arm early prospective phase I study	Lymphadenectomy followed by SBRT/SRS	SBRT/SRS	Y	10	Portion of patients with ≥ Grade 3 treatment-related toxicities	March 2013

* = based on treatment center or treating medical oncologist discretion. When available, the dose used for SBRT/SRS is indicated between parentheses as dose in Gray (Gy) / Number of fractions (F). PFS = Progression-free survival. OS = Overall survival. RR = Response rate. ORR = Objective response rate.

**Table 3:** Ongoing Clinical Trials for Oligoprogressive NSCLC.

Study Title and Number	Treated Condition	Oligoprogressive Definition	Study Design	Treatment Scheme	SBRT/SRS	Surgical Excision	Number of Participants	Primary Outcome	Start Date
SBRT for Oligoprogressive NSCLC After First Line Treatment With Immune Checkpoint Inhibitors NCT05387044	Pathologically confirmed oligoprogressive NSCLC after first line treatment with immune checkpoint inhibitors	1–5 in 1–3 organs and max 5 cm.	Single-arm prospective phase II study	SBRT/SRS	SBRT/SRS (N/A)	N	28	PFS	May 2022
SBRT for Oligoprogressive NSCLC After Treatment With PD-1 Immune Checkpoint Inhibitors NCT04767009	Pathologically confirmed oligoprogressive NSCLC after treatment with PD-1 inhibitors	Not provided.	Single-arm prospective phase II study	SBRT/SRS *	SBRT/SRS (N/A)	N	59	Percentage of participants with adverse events	January 2021
								1-year new lesion-free survival rate	
Atezolizumab Plus 8 Gy Single-fraction Radiotherapy for Advanced Oligoprogressive NSCLC NCT04549428	Histologically or cytologically confirmed oligoprogressive NSCLC after anti PD-1 agent and first-line of chemotherapy	1–4 in 1–3 organs. 1–3 in 1 organ.	Single-arm prospective phase II study	Atezolizumab intravenously followed by SBRT/SRS	SBRT/SRS (8 Gy/1 F)	N	20	ORR	October 2020
LAT for Oligoprogressive NSCLC Treated With First-line OSImertinib (LAT-FLOSI) NCT04216121	Histologically confirmed EGFR mutated oligoprogressive NSCLC not suitable for radical treatment after first-line TKI therapy with osimertinib	1–3	Prospective observational study	SBRT/SRS followed by surgery	SBRT/SRS (N/A)	Y	39	PFS 2	May 2021
A Study of Furmonertinib Combined With Radiotherapy for Non-small Cell Lung Cancer With Oligoprogression NCT04970693	Histologically or cytologically confirmed oligoprogressive NSCLC after first-line EGFR-TKI therapy	3–5 after receiving the first or second generation of EGFR-TKI or after receiving Osimertinib and chemotherapy was refused.	Single-arm prospective phase II study	Furmonertinib in combination with local radiotherapy	N	N	64	PFS	June 2021
Local Consolidative Therapy and Durvalumab for Oligoprogressive and Polyprogressive Stage III NSCLC After Chemoradiation and Anti-PD-L1 Therapy NCT04892953	Oligoprogressive or polyprogressive NSCLC and received standard chemoradiation and anti-PD-l1 durvalumab therapy.	1–3	Two-arm prospective phase II study	Local consolidative therapy in combination with Durvalumab intravenously	N	Y *	51	PFS	July 2021
				Local consolidative therapy in combination with Durvalumab intravenously and chemotherapy					
Local Ablative Therapy for Treatment of Oligoprogressive, EGFR-Mutated, Non-Small Cell Lung Cancer After Treatment With Osimertinib NCT02759835	(1) Histologically confirmed advanced lung adenocarcinoma with EGFR-sensitizing somatic mutations or a germline T790M mutation with no prior EGFR TKI therapy. (2) Progressive disease after 1st or 2nd generation EGFR TKI therapy harboring somatic T790 M mutation. (3) Progressive disease after treatment with Osimertinib who are eligible for local ablative therapy.	Not provided	Two-arm prospective phase II study	Osimertinib followed by LAT * followed by Osimertinib	N	Y *	37	PFS 2	April 2016
				LAT * followed by Osimertinib				PFS	
Stereotactic Ablative Radiotherapy for Oligo-Progressive Non Small Cell Lung Cancer (SUPPRESS-NSCLC) NCT04405401	Oligoprogressive NSCLC while on ICI or TKI	1–5 extracranial in 1–3 organs and max. 5 cm	Two-arm prospective phase II study	Systemic therapy *	SBRT (N/A)	N	68	PFS	January 2021
				SBRT in combination with systemic therapy *				OS	
Stereotactic Body Radiotherapy for the Treatment of OPD (HALT) NCT03256981	Histologically confirmed mutation positive advanced oligoprogressive NSCLC following initial response to a TKI	1–3 extracranial	Two-arm prospective phase III study	Continued TKI therapy only	SBRT (N/A)	N	110	PFS	November 2017
				Continued TKI therapy in combination with SBRT					
Randomized Study of Stereotactic Body Radiation Therapy (SBRT) in Patients With Oligoprogressive Metastatic Cancers of the Breast and Lung NCT03808662	Histologically confirmed triple negative breast cancer, or NSCLC (without known EGFR mutation or ALK/ROPS1 rearrangement), or other high-risk breast cancer progressed on hormone or systemic therapy, or NSCLC with EGFR, ALK, or ROS1 targetable molecular alterations with disease progression on first line TKI.	1–5 in 1 extracranial organ	Two-arm prospective phase II study	Standard medical treatment *	SBRT/SRS (*)	N	107	PFS	January 2019
				SBRT/SRS					

* = based on treatment center or treating medical oncologist discretion. When available, the dose used for SBRT/SRS is indicated between parentheses as dose in Gray (Gy) / Number of fractions (F). PFS = Progression-free survival. OS = Overall survival. ORR = Objective response rate.

## Abbreviations

All abbreviations were defined the first time they appear in each of article section. ESTRO: European Society of Radiation Oncology; ASTRO: American Society of Radiation Oncology.
